# On Using a Mobile Application to Support Teledermatology: A Case Study in an Underprivileged Area in Colombia

**DOI:** 10.1155/2018/1496941

**Published:** 2018-03-26

**Authors:** Juan Pablo Sáenz, Mónica Paola Novoa, Darío Correal, Bell Raj Eapen

**Affiliations:** ^1^Dipartimento di Automatica e Informatica, Politecnico di Torino, Turin, Italy; ^2^Fundación Universitaria de Ciencias de la Salud, Hospital de San José, Bogotá, Colombia; ^3^Departamento de Ingeniería de Sistemas y Computación, Universidad de los Andes, Bogotá, Colombia; ^4^McMaster University, Hamilton, ON, Canada

## Abstract

**Background:**

The use of mobile applications in dermatology to support remote diagnosis is gaining acceptance, particularly in rural areas, where dermatology services are commonly managed by healthcare personnel with no specialty training. Moreover, ontologies—sets of concepts that represent knowledge in a given domain—are increasingly being used to support medical diagnosis. A specific case is ONTODerm: an ontology to aid dermatological diagnosis. However, there is little information on the combined use of mobile applications and ontologies as support solutions in dermatology.

**Objective:**

Assessing the reliability of ONTODerm as a tool to support remote dermatological diagnosis when used together with a mobile dermatological application in underprivileged areas.

**Methods:**

A mobile application that allows characterization of skin lesions was developed, and the information about the lesions was sent to ONTODerm. An exploratory study was conducted in a remote area without access to a dermatologist. A total of 64 dermatological queries were recorded in the application and consulted with ONTODerm. Later, an experienced dermatologist evaluated the characterization and diagnosis of each query to determine the accuracy of the system.

**Results:**

The results showed that the probability of obtaining a correct diagnosis was between 64.4% and 85.6% with a confidence interval of 95%. A higher accuracy rate was obtained when the skin lesion occurred on the face or when its border was categorized as poorly demarcated.

**Conclusions:**

This study demonstrates the implementation of a teledermatology strategy based on mobile applications and domain ontology-driven knowledge base to provide timely assistance to healthcare professionals. This approach was found to be pertinent in the Colombian rural context, particularly in forest regions, where dermatology specialists are not available. The results of this article do not represent a final validation of the proposed approach; they suggest how the ontology can be improved to effectively support medical staff in marginalized regions.

## 1. Introduction

The use of mobile health applications (or mHealth) to support telemedicine is becoming widely adopted. The ease of use and the ubiquity of mobile phones make their use in remote diagnostic applications increasingly viable [1–3].

Mobile applications are also popular in teledermatology [4–8]: the use of telecommunication technologies to support the diagnosis and treatment of skin conditions. These applications are particularly relevant in remote locations, where the possibility of finding a dermatology practice is low. In Colombia, according to the Colombian Association of Dermatology, there were 1.25 dermatologists per 100,000 inhabitants in 2011, with a large number of them in larger and more densely populated cities (3 per 100,000 inhabitants), thus leaving rural areas without specialized dermatological care options [9].

In rural areas, dermatological services are commonly provided by medical staff without dermatology expertise. Consequently, complex queries are sent to specialists by email, and the answers may take several days to arrive. Under these circumstances, the use of mobile applications to enable real-time dermatological diagnosis (in a rural context) has a great potential in countries like Colombia [10].

### 1.1. Ontologies

An ontology is a model of a particular domain, built for a particular purpose [11]. Computational ontologies are a means to formally model the structure of a system through the relevant entities and relations that emerge from its observation [12]. When domain knowledge is formally declared, it is possible to make explicit inferences about the concerned domain and get a common understanding of the concepts that are modeled in the ontology. The relevance of ontology in the health sector is described by Kiong et al. [13]. The authors present the development of a health ontology system aimed at integrating relational data (stored in the form of databases) with a set of concepts and their associated semantics. This integration would enable the machines to make interpretations over that information through semantic agents for healthcare domain. Likewise, Maragoudakis et al. [14] describe an approach to the use of ontologies for reducing the ambiguity of dermatological terms. Through a domain ontology, authors attempt to standardize the protocols and terminology in the domain of skin lesions, facilitating a better understanding of the symptoms and development of early skin cancers.

ONTODerm [15] is an ontology for dermatology that was designed to be collaboratively developed by domain experts to analyze, modify, and visualize the data in a convenient and accurate manner, without the need for technical instructions. DKB is a Resource Description Framework (RDF) based knowledge base built using ONTODerm. DKB offers four main features: (I) describing the characteristics of a given disease, (II) identifying the differential diagnosis for a given set of features, (III) listing the differential diagnoses of a given disease, and (IV) enumerating the synonyms of a given disease.

Our study made use of the second feature. DKB receives a set of terms corresponding to the characterization of a skin lesion (i.e. “women, macule, multiple, confluent, poorly demarcated, and face”) and, making use of its inference ability, determines a set of possible differential diagnoses and retrieve them in a list. For example, in the above query, the ontology returns the following result: “ChloasmaFaciei 46, Melasma 46, Ephelides 46, Freckles 46, Lichenoid Keratosis 46, Mask Of Pregnancy 46, Benign Lichenoid Keratosis 46, Lupus Panniculitis 36.”

The associated number is an arbitrary scale for the degree of match and can be used by external applications to prioritize the arrangement.

### 1.2. Teledermatology

Several proposals concerning mobile devices in dermatology have already been developed [16]. In fact, mobile teledermatology is established as a technically feasible and diagnostically reliable method of increasing access to dermatologic expertise in poorer regions of the world, where access to computers with Internet is unreliable or insufficient. Azfar et al. [17] demonstrated that HIV-positive patients, in a resource-limited setting in Botswana, find mobile teledermatology acceptable for specialist consultations when face-to-face consultations may be difficult to obtain. Ebner et al. [18] and Schreier et al. [19] present two successful mobile teledermatology approaches that rely on sending mobile phone camera images to a dermatologist. However, to the best of our knowledge, studies reporting the use of ontologies and mobile applications for supporting teledermatology are scarce.

**Figure 1:**
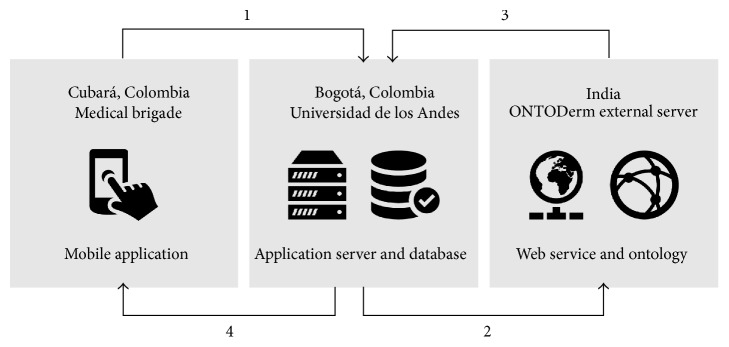
Skinhealth overall perspective view.

In this article, we present Skinhealth, a system that supports the diagnostic process of skin lesions by using an ontology and a knowledge base system integrated with a mobile application (1). This application was developed conjointly by the Systems and Computing Engineering Department at Universidad de los Andes (DISC) and Colombian dermatologists. Skinhealth is composed of a client application and a server application that runs on the DISC.  Figure 1 presents a schematic view of the proposed solution.

**Figure 2:**
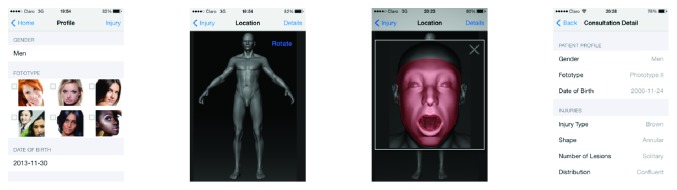
Screenshots of the mobile application.

To illustrate our proposal, let us consider the process presented in Figure 1. In step one, the healthcare professional in a remote area uses the Skinhealth mobile application to characterize a dermatologic lesion according to a fixed set of parameters. The mobile application then connects to an application server located in the DSIC in Bogotá and transfers the data. In step two, the application server transfers the query to ONTODerm using a web service hosted on an external server (http://gulfdoctor.net/dermbase). Once the external web service receives the request, it formulates a query to ONTODerm. In step three, the web service returns eight differentials to the application server in Bogotá. In step four, the application server stores the differentials obtained for the query and sends them to the mobile app in the rural area. This information is used by the healthcare professional during the diagnosis process.  Figure 2 presents the mobile application used by the healthcare professional in the remote area.

### 1.3. Health Brigades

This paper reports the results of the first-time use of Skinhealth in a rural area in Colombia [20]. The use of the app was made within the context of a program called Health Brigades in Colombia. Health brigades have been carried out since 2003 and are organized by the foundation “Alas Para la Gente” (Wings for the People) [21]. These brigades have dozens of volunteering doctors and support of the Colombian Air Force and the Colombian military since they are usually conducted in remote areas with potential public order problems.

### 1.4. Objective

The objective of this study is to identify the pertinence and accuracy of ONTODerm and DermKnowledgeBASE when used as a tool to support remote dermatological diagnosis, in conjunction with a mobile dermatological application in a rural environment.

## 2. Methods

A health brigade conducted in a rural area in Colombia without immediate access to a dermatologist was selected as the scenario to analyze the ability of Skinhealth and ONTODerm in supporting remote diagnosis of dermatological lesions. The study population included all patients who attended the brigade without any exclusion criteria.

The study was conducted in two stages. The first stage involved a general physician in the health brigade (with no special training in dermatology) and an assistant in charge of the Skinhealth mobile app. At every visit, the physician characterized the skin lesions according to a set of parameters. These parameters and the possible values that they could assume are presented in Table 1. The characterization of the skin lesion made by the physician, along with his suggested diagnosis, was registered by the assistant in the mobile application. Then, this characterization was submitted to the main server, in Bogotá, which invoked the DKB web service. The response from the web service was forwarded from the main server to the mobile application. Finally, the assistant verified that Skinhealth received and stored the result given by DKB, and the visit is completed. It is important to note that the general practitioner neither knows nor has access to the results sent by DKB to avoid being influenced by the ontology.

The second stage was performed once the health brigade concluded. A dermatologist with broad experience in skin diseases examined the characterization of each query and compared this information with the diagnosis made by the general practitioner, as well as with the results obtained from the web service. Based on this review, each diagnosis was classified as accurate or inaccurate, for both the diagnosis given by the general physician and the differentials obtained from the web service. The physician's diagnosis was marked as accurate if it matched with the dermatologist's diagnosis. In the case of the ontology, the set of eight differentials was marked as accurate if at least one of the differentials was consistent with the dermatologist's diagnosis.

### 2.1. Outcomes Measures

This study measured five characteristics that we considered important in determining the behavior of Skinhealth and ONTODerm. These measurements are presented below.

#### 2.1.1. Demographics

The demographic information of the patients treated in the brigade is presented for the purpose of illustrating the general characteristics of the population addressed.

#### 2.1.2. Percentage of Accuracy

The second measure is the percentage of correct diagnoses retrieved by the knowledge base; it indicates the proportion of queries in which the differentials were accurate given a certain lesion characterization. Confidence intervals were used to establish a more objective measure.

#### 2.1.3. Evaluation of Results

The third measure accounts for the accuracy of the ontology by identifying those cases in which the ontology has better performance and those in which it fails to suggest accurate differentials. To calculate this indicator an inferential statistical analysis was performed recognizing the influence that the values of certain parameters have on the accuracy of a query. We believe that the observations arising from this measure will help understand the circumstances under which the ontology can be improved.

#### 2.1.4. Filtering Based on Inferential Analysis

Based on the results of the inferential analysis, the values that negatively affected the accuracy of the differentials retrieved by DKB were excluded from the queries.

#### 2.1.5. Specialist's Feedback

The fifth measure categorizes the reasons why certain queries, according to the expert, were classified as not accurate; thus, this measure provides some considerations to improve the precision of the ontology.

#### 2.1.6. General Physician's Diagnostic Accuracy

Considering that the app is portrayed as a support tool for general practitioners, this measure describes the cases in which the lesion characterization made by the general practitioner was not consistent with his suggested diagnosis.

### 2.2. Statistical Analysis

A descriptive statistical analysis of the results was performed to identify the demographic characteristics of the population and the most frequent values in the characterization of the lesions. Subsequently, in order to draw conclusions regarding these characterizations, an inferential statistical analysis was conducted. The purpose of the inferential analysis was to determine which parameter values influence positively an accurate diagnosis and against which values the ontology is more likely to yield inaccurate results. In this inferential analysis, confidence intervals were applied to the parameters in which the sample size was big enough. Therefore, the results of the inferential analysis help to identify the expected behavior from the specific population where the brigade was carried out.

## 3. Results

Phase 1 of this study was conducted on the 26, 27, and 28 July 2013 in Colombian municipality Cubará, which has a population of 3,118 inhabitants: 1,551 women and 1,567 men, most of them belonging to the U'wa indigenous tribe. The nearest medical center with a dermatologist available is 166 kilometers away in Cúcuta city, at an eight-hour overland journey through a jungle area.

During the three-day brigade, 895 medical consultations were performed, distributed as follows: optometry, 18.43%  (*n* = 165), general medicine, 14.18%  (*n* = 127), pediatrics, 14.07%  (*n* = 126), internal medicine, 9.72%  (*n* = 87), alternative medicine, 8.04%  (*n* = 72), gynecology, 7.26%  (*n* = 65), dermatology, 7.26%  (*n* = 65), orthopedics, 6.25%  (*n* = 56), dentistry, 5.92%  (*n* = 53), veterinary, 4.69%  (*n* = 42), vaginal cytologies, 3.46%  (*n* = 31), and outpatient surgeries, 0.67%  (*n* = 6). Medical services were provided in the health center and in classrooms of the village school. A general physician attended dermatological visits between 8 am and 5 pm. Skinhealth was installed on a tablet with version 4.2 of the Android OS.

### 3.1. Demographic Information

The number of men who attended the dermatological consultation (*n* = 20) was less than half the number of women (*n* = 44), and the standard deviation of the averages (*n* = 17) and ranges of ages accounted for the demographic diversity of patients evaluated during the brigade. The most common skin phototypes were III (*n* = 24) and IV (*n* = 23). There were no people with skin phototypes I or VI.

Most women were between 15 and 38 years old, whereas most males were between 15 and 50 years old. The number of patients below the age of 12 or older than 60 years was small, comprising only eighth of the total number of patients (Table 2).

### 3.2. Ontology's Percentage of Accuracy

Phase 2 of the study took place in Bogotá two weeks after the brigade. In this phase, the dermatologist followed the established validation protocol; namely, she analyzed the values given by the general practitioner for each parameter of Table 1 during phase 1. The characterizations of the lesions were validated along with the diagnosis given by both the general physician and the ontology, and each query was assessed by the dermatologist as accurate or inaccurate.

In 75%  (*n* = 48) of the cases, the ontology provided accurate results.  Table 3 presents the point estimate and three confidence intervals for the entire sample segmented by gender.

**Figure 3:**
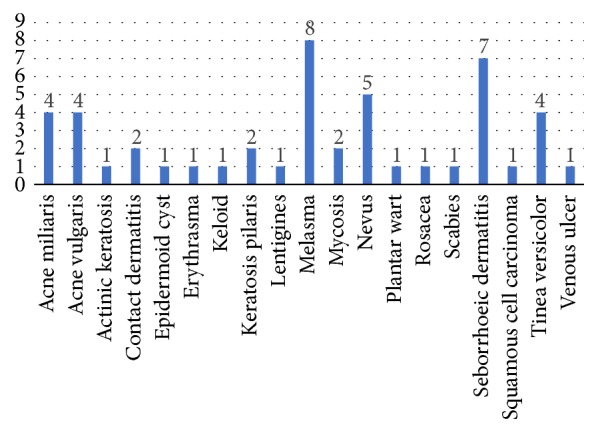
Diagnoses of the consultations rated as accurate.

**Figure 4:**
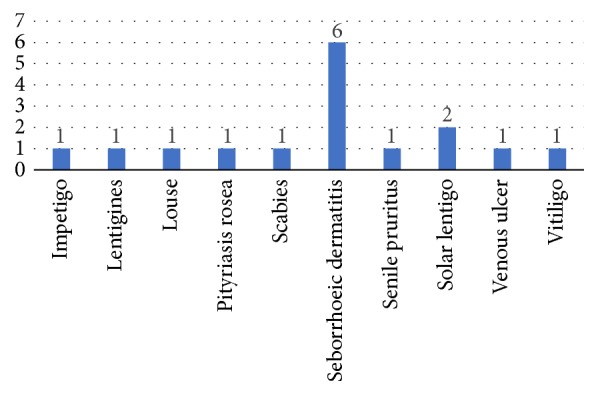
Diagnoses of the consultations rated as not accurate.

Coincidentally, the point estimate of the proportion of correct diagnoses in men and women was the same. It should be noted that the sample size for men is not large enough; thus, confidence intervals are very broad and do not provide accurate information. In contrast, confidence intervals for women are smaller and allow the formulation of more precise observations.  Table 3 shows that there is no difference between the proportions of correct diagnoses in men and women.  Figure 3 presents the diagnoses of the consultations rated as accurate, while Figure 4 presents diagnoses of the consultations rated as not accurate.

### 3.3. Evaluation of Results

We noticed some interesting observations regarding “affected areas,” “border,” and “type of lesion” as detailed below.

#### 3.3.1. Affected Areas

As can be seen in Table 4, most skin lesions were identified in the face and hair, comprising 60% of total observations. Since the volume of pooled data was high, it was possible to separately analyze the queries of lesions on the face and the queries of lesions on the hair in order to determine the degree of accuracy in each case.

The percentage of accurate queries in which the face was identified as the affected area was 96%. It was noted that, for “face,” the values of other parameters were not recurrent. The above shows that, regardless of the value of the other parameters, the predictive power of web service is favorable when the lesion occurs on the face.

In contrast, when the affected area is the hair, the inaccuracy of the results cannot be explained not only due to the affected area but also due to other parameters that were recurrent among these queries involving dermatologic lesions in the hair area. A special case is one in which the affected area is the hair; the lesion is patch-type, the number is solitary, the distribution is asymmetrical, and the shape is annular.

#### 3.3.2. Border

The ontology showed greater accuracy for those lesions with an indistinct border. An individual analysis of the behavior of queries by type of demarcation showed that 81.40% of poorly demarcated lesions were correctly identified by the ontology, while the percentage of those well demarcated was 61.90%.

When excluding queries in which the affected area was the hair or face, 85.71% of ill-demarcated lesions were accurate, while in well-demarcated lesions this percentage was 58%. Similarly, we analyzed the queries with strong demarcation and inaccurate validation and determined that there is no second feature repeated in these consultations. This allows us to infer that the system is less accurate when the lesion is well demarcated.

In contrast, we found that when lesions with ill-demarcated border were inaccurate, it also occurred that most of them corresponded to the case that was identified by analyzing the affected areas; that is, in the case in which the affected area was the hair, the lesion was patch-type, the number was solitary, the distribution was asymmetric, and the shape was annular. Thus, the failure in accuracy could not be attributed to the fact that the lesion was poorly demarcated.

This observation leads to the conclusion that the ontology performs better in cases in which the demarcation is indistinct, except for the case mentioned above.

#### 3.3.3. Distribution of the Lesion

When the distribution of the lesion was asymmetric, differentials provided by the ontology were deemed accurate in approximately 50% of cases. However, when the distribution was not asymmetric, this percentage increased to 85% of cases. Hence, the system performs better for asymmetrically distributed lesions.

#### 3.3.4. Special Case

From inferential analysis of parameters such as the affected area, border, and distribution of the lesion, we identified a particular case in which a precise combination of values generated a set of differentials categorized as inaccurate. In this specific case, the affected area was the hair, the lesion was patch-type, the number was solitary, the distribution was asymmetric, and the shape was annular. It occurred 11 times and accounted for 37.50% of queries catalogued as inaccurate. These incidences correspond to inquiries in which the general practitioner diagnosed seborrheic dermatitis. Just in two cases, the diagnosis of seborrheic dermatitis coincided with the differentials suggested by the ontology.

#### 3.3.5. Other Parameters

Other parameters were not analyzed individually because the ontology did not display performance problems in any case. Similarly, it was determined that inaccurate cases were mainly associated with a strong demarcation, an asymmetric distribution, or the particular case described above.

We performed an analysis of the sample excluding the queries with these values that seemingly had a negative impact on the accuracy.  Table 5 presents the proportion of accurate queries and the probability of obtaining an accurate result with a 95% confidence interval.

*Special Case (SC).* When queries corresponding to the special case were subtracted from the sample (*n* = 53), the point estimate of the proportion of accurate queries increased from 75% to 81%, and the probability of obtaining an accurate result with the ontology fluctuated between 70.6% and 91.7%.

*Strong Demarcation and Special Case (SD).* If, furthermore, lesions with strong demarcation are excluded (*n* = 32), the proportion of accuracy in the point estimate rises to 94% with a probability greater than 85%.

*Asymmetrical Distribution (AD).* On the other hand, when lesions with asymmetric distribution are excluded, the point estimate of the proportion of accurate queries rises to 88%, with a probability between 77.85% and 97.8%.

### 3.4. Feedback from Specialist

The dermatologist provided feedback on the reasons why the differentials supplied by the ontology for certain queries were classified as inaccurate. The reasons for rejection of results were categorized as follows.

*Age.* The diagnoses suggested by the ontology represented diseases that do not occur in the age range of the patient.

*Gender.* The diagnoses suggested by the ontology represented diseases that do not occur in people of the patient's gender.

*Accuracy in the Affected Area Selection.* The degree of specificity of ontology in relation to areas of the body was not enough for the suggested diagnoses to be related to the patient's affected area.

*Type of Lesion.* The suggested diagnoses do not correspond to the type of lesion identified in the characterization.

**Figure 5:**
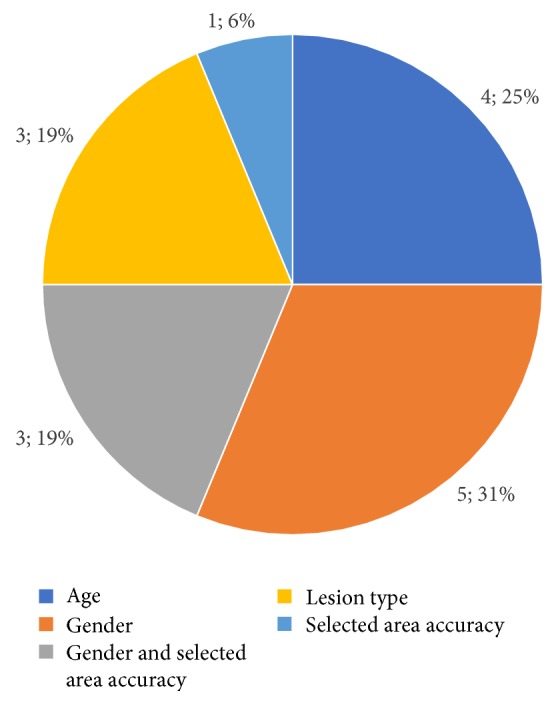
Frequency of referral to previously stated reasons for declaring a query inaccurate.

Figure 5 summarizes the frequency of referral to previously stated reasons for declaring a query as inaccurate.

### 3.5. Level of Accuracy of the General Physician

An experienced dermatologist determined that, in eight queries, characterization of the lesion was not consistent with the diagnosis submitted by the general practitioner. Of these eight queries, the ontology tossed accurate results in six of them, despite incorrect characterization.

## 4. Discussion

At first, without classifying or segmenting results, the ontology provides favorable diagnoses with a probability between 64.4% and 85.6% (95% confidence interval), regardless of patient and lesion characteristics. Similarly, it was determined that there is no statistical difference concerning the effectiveness of the program when the patient is male or female; in both cases, the program gave positive results for 75% of queries.

Regardless of other parameters, ontology had great accuracy when the area of the lesion was the face. In contrast, results were more likely to be inaccurate when the lesion's borders were well demarcated and had asymmetric distribution.

We identified a particular case in which, irrespective of whether borders were weakly demarcated, the results were not favorable. When excluding queries with strong demarcation and those corresponding to the particular case previously stated, the effectiveness of the program increased significantly.

### 4.1. Limitations

All the statistical analysis was performed on a sample of 64 dermatologic consultations. It should be remarked that the conclusions drawn from this sample can be generalizable just to the population from which the sample was extracted, notwithstanding that a sample size greater than 30 was considered high enough for cases in which inferential statistics are carried out. During the development of this study, we were limited to the number of dermatology patients attending the brigade. We are aware that the size of the sample is small, and it is necessary to use Skinhealth in future medical brigades to validate our findings.

## 5. Conclusions

In the present study, we proposed the integration of a mobile app and its respective connectivity and portability features with the ability of inference and learning of a dermatological ontology. We tried to ascertain the potential of this solution in dermatological diagnostic work carried out by general practitioners in rural and geographically marginalized municipalities, where there are no specialists.

Overall, this study represents the first controlled evaluation of a teledermatology strategy that relies on mobile applications and domain ontology to immediately assist a general practitioner who answers dermatological consultations. This approach was found to be relevant in the Colombian context, particularly in geographically and economically marginalized regions. We believe that the results help to understand the circumstances under which the ontology can be improved to effectively support the general practitioners in the diagnosis of dermatologic lesions. However, since the sample size is small, more studies would be necessary to validate the findings. The conclusions drawn in this paper do not represent a final validation of Skinhealth but a preliminary evaluation to determine its potential as a tool that can support medical staff in regions where there are no dermatology specialists.

## Figures and Tables

**Table 1:** The parameters that the application takes into account to describe dermatologic lesions.

Parameter	Possible values
Birth date	—
Sex	Female, male
Phototype (Fitzpatrick scale)	Phototype I, phototype II, phototype III, phototype IV, phototype V, phototype VI
Lesion type	Atrophic, cyst, macule, nodule, patch, plaque, pustule, ulcer, vesicle
Shape	Annular, circinate, dome-shaped, ragged, oval, pedunculated, rounded, umbilicated
Lesion number	Disseminated, multiple, recurrent, solitary
Lesion distribution	Asymmetrical, confluent, scattered, symmetrical
Affected areas	Abdomen, genital, arm, dorsal, buttocks, chest, foot, hand, ears, face, finger, hand, nail, finger, head, leg, neck, palmar, hair, plantar, finger, foot, nail
Border	Poorly demarcated, well demarcated
Appearance date	—
Symptoms	Alopecia, blanching, desquamation, pain, edema, eruption, excoriation, exfoliation, hemorrhage, pigmentation, pruritus, fever, facial paralysis, weight loss, systemic symptoms
Past	Anemia, arthritis, malnutrition, diabetes, epileptic, hypertension, hypotension, myocarditis, neuropathy

**Table 2:** Demographic information.

Sex	*n*	*%*
Male	20	31
Female	44	69
Total	64	100
Age	Average (years)	SD
Male	38	16
Female	31	19
Total	33	17
Age range	Minimum age	Maximum age
Male	15	83
Female	2	74
Total	2	83
Phototype (Fitzpatrick scale)	*n*	*%*
Phototype I	0	0
Phototype II	16	25
Phototype III	24	37
Phototype IV	23	36
Phototype V	1	2
Phototype VI	0	0
Total	64	100

**Table 3:** Percentage of accurate results.

	Total	Male	Female
Sample size (*n*)	64	20	44
Point estimate	0.75	0.75	0.75
95% CI	[0.644; 0.856]	[0.560; 0.940]	[0.622; 0.878]

**Table 4:** Most frequent values for each parameter class.

	*n*	%	Accurate (%)	Not accurate (%)
Lesion type				
Patch	21	32.8	14 (66.6)	7 (33.3)
Macule	12	18.7	9 (75.0)	3 (25.0)
Total	33	51.5		
Number				
Solitary	26	40.6	17 (65.4)	9 (34.6)
Multiple	23	25.9	19 (82.6)	4 (17.4)
Total	49	76.5		
Shape				
Annular	18	28.2	10 (55.5)	8 (44.5)
Ragged	14	21.8	11 (78.6)	3 (21.4)
Total	32	50		
Distribution				
Asymmetrical	23	35.9	12 (52.2)	11 (47.8)
Confluent	13	20.3	11 (84.6)	2 (15.4)
Total	36	56.2		
Border				
Poorly demarcated	43	67.1	35 (81.4)	8 (18.6)
Total	43	67.1		
Affected areas				
Face	25	39.0	24 (96.0)	1 (4.0)
Hair	13	20.3	7 (53.8)	6 (46.2)
Total	38	59.3		

**Table 5:** Results obtained when the Special Case (SC), the Strong Demarcation and Special Case (SD and SC), and the Asymmetrical Distribution (AD) were excluded.

	SC	SC and SD	AD
Consultations (*n*)	53	32	41
Certitude (%)	81.0	94.0	88.0
95% CI	[0.706; 0.917]	[0.85; 1.0]	[0.788; 0.978]

## Abbreviations

DISC: Departamento de Ingeniería de Sistemas y Computación
DKB: DermKnowledgeBASE.
